# Machine Learning-Based Prediction of Readmission Risk in Cardiovascular and Cerebrovascular Conditions Using Patient EMR Data

**DOI:** 10.3390/healthcare12151497

**Published:** 2024-07-28

**Authors:** Prasad V. R. Panchangam, Tejas A, Thejas B U, Michael J. Maniaci

**Affiliations:** 1 Data Science Team, Saigeware Inc., Karnataka 560070, India; tejas@saigeware.com (T.A.); thejas.bu@saigeware.com (T.B.U.); 2 Enterprise Physician Lead, Advanced Care at Home Program, Mayo Clinic Hospital, Jacksonville, FL 32224, USA; maniaci.michael@mayo.edu

**Keywords:** comorbidities, EMR data, LACE plus score, risk of readmission, XGBoost

## Abstract

The primary objective of this study was to develop a risk-based readmission prediction model using the EMR data available at discharge. This model was then validated with the LACE plus score. The study cohort consisted of about 310,000 hospital admissions of patients with cardiovascular and cerebrovascular conditions. The EMR data of the patients consisted of lab results, vitals, medications, comorbidities, and admit/discharge settings. These data served as the input to an XGBoost model v1.7.6, which was then used to predict the number of days until the next readmission. Our model achieved remarkable results, with a precision score of 0.74 (±0.03), a recall score of 0.75 (±0.02), and an overall accuracy of approximately 82% (±5%). Notably, the model demonstrated a high accuracy rate of 78.39% in identifying the patients readmitted within 30 days and 80.81% accuracy for those with readmissions exceeding six months. The model was able to outperform the LACE plus score; of the people who were readmitted within 30 days, only 47.70 percent had a LACE plus score greater than 70, and, for people with greater than 6 months, only 10.09 percent had a LACE plus score less than 30. Furthermore, our analysis revealed that the patients with a higher comorbidity burden and lower-than-normal hemoglobin levels were associated with increased readmission rates. This study opens new doors to the world of differential patient care, helping both clinical decision makers and healthcare providers make more informed and effective decisions. This model is comparatively more robust and can potentially substitute the LACE plus score in cardiovascular and cerebrovascular settings for predicting the readmission risk.

## 1. Introduction

Hospital readmissions can significantly disrupt the daily lives of patients’ families and caregivers, leading to potential harm for the patients, a decrease in the quality of care [1,2], and an overall increase in healthcare costs. Nearly twenty percent of the patients in the US are prone to readmission within 30 days after their initial discharge [3,4]. The hospital readmission rate is a key metric used to evaluate the quality of care provided by a hospital [5,6]. Additionally, efforts to reduce avoidable hospital readmissions have the potential to alleviate both the financial and healthcare burden while enhancing the quality of the care [7,8]. The development of an algorithm designed to assess and predict this risk holds the potential to greatly assist caregivers in understanding each patient’s unique situation. By leveraging such an algorithm, caregivers can proactively identify patients at higher risk of readmission, enabling early interventions and targeted measures to mitigate that risk. This not only enhances the quality of care but also contributes to better resource allocation within healthcare facilities. Ultimately, understanding and quantifying readmission risk via advanced algorithms empowers caregivers to make more informed decisions and deliver more effective patient-centered care.

The predictive modeling of hospital readmission risk has been gaining popularity in the field of medical science. Scores like LACE [9,10] and HOSPITAL [11] have been widely used to understand the risk of readmission. These scores have used the length of stay, patients’ emergency history, lab results, and comorbidities to calculate the risk scores. Since the calculation of risk is more complex and many factors influence it, ML [12,13,14] has now been applied to solve these problems. ML can capture more complex non-linear correlations and can be easily applied to very high-dimensional data [15]. The application of machine learning (ML) techniques in predicting the risk of readmission has been a burgeoning area of research, with various approaches adopted by different studies. Many of these studies have traditionally relied on fundamental patient information, including age, gender, race, length of stay, and basic lab results [12,16]. While this approach has yielded positive results, it often falls short of incorporating crucial patient-specific factors such as comorbidities and medication history, which are known to significantly impact readmission risk [13,17]. Most of the studies and research conducted only include one or a few aspects of the above-mentioned patient-related information. As a result, they often fail to comprehensively understand and predict the risk of patient readmission.

In order to address these issues, we propose a novel approach to determine the number of days to readmission. The EMR data of the patient are taken from the EHR database. The data consist of lab details, vitals, medications, comorbidities, and the admit–discharge settings. The data are then processed and a machine learning algorithm is used to predict the risk of readmission. This study takes inspiration from a 30-day readmission risk model developed by [12], which utilized a sizable dataset of 100,000 patient records. Although the study [12] proved to be a landmark work, it is limited to predicting readmission within a specific timeframe. Our study tries to predict the risk of readmission by predicting the ranges between which a patient may be readmitted.

The study involved the evaluation of a wide range of significant laboratory test outcomes, which offered insightful information about the physiological state of the patients. These lab tests comprised ECG (electrocardiogram), serum sodium, serum potassium, hemoglobin, blood glucose, and blood urea nitrogen. Each of these tests provided crucial information for the comprehensive evaluation of the patients’ health status. The study also took inspiration from works [12] that pinpointed a number of crucial elements affecting the HOSPITAL scores [18]. The key features in this regard encompassed the lab sodium levels, hemoglobin levels, type of admission, past admissions within the preceding 12 months, and length of stay. These characteristics were discovered to be important predictors in assessing and predicting the probability of hospital readmissions. In the context of stroke and cardiovascular disorders, studies [19,20,21] have emphasized the crucial significance of two important laboratory markers, serum potassium and blood glucose levels. They have demonstrated a strong and significant correlation between these two parameters and the occurrence and progression of these debilitating health conditions. So, our study includes lab results as one of the primary markers in identifying the risk of readmission.

Vital signs are pivotal indicators in the realm of healthcare as they provide critical insights into a patient’s condition both at the time of admission and with regard to their future prognosis [22]. The dynamics and variations in vital signs throughout a patient’s stay can be considered as essential pieces of the puzzle for assessing the individual’s risk of readmission. A comprehensive study [22] was conducted to elucidate the intricate relationship between unstable vital signs at the time of discharge and the likelihood of readmission over a one-year time frame. The results of this investigation underscored a direct and substantial correlation between instabilities in vital signs within the 24 h prior to discharge and an increased seven-day readmission rate. This finding underlines the importance of closely monitoring and interpreting vital signs as part of the discharge process.

A study [13] was undertaken to understand the risk factors for hospital readmission in older adults within a 30-day timeframe. The findings of this research revealed a direct and impactful correlation between the quantity of medications prescribed to a patient during their stay and their susceptibility to readmission. Also, the significance of the diagnosis information in comprehending a patient’s risk cannot be overstated. In our study, we consistently incorporated both the primary diagnosis code and the diagnosis codes that were present upon admission. These diagnostic codes are instrumental in providing comprehensive and detailed insights into the severity of a patient’s medical condition at the time of the index admission. The demographic data of a patient, such as race, gender, and age, alongside clinical factors like admission type, source of admit, length of stay, and previous hospital visits, are also key in identifying the risk of readmission.

The choice of the model used for training had a major impact on the study. The XGBoost algorithm [23], a potent Gradient Boosted Decision Tree (GBDT) [24] machine learning technique, served as the foundation for training the predictive model. XGBoost, a cutting-edge ensemble learning technique, combines the strengths of decision trees and gradient boosting, making it an ideal choice for complex prediction tasks. The effectiveness of GBDT algorithms like XGBoost in predicting readmissions has been shown in [25,26]. In fact, it has been demonstrated that these algorithms perform on par with or even better than many deep learning approaches [13,14,16,17], which makes them ideally suited for addressing the challenges in predicting readmission. For model training, XGBoost was used. In the subsequent sections, we will describe the methodology employed and results obtained from this study.

## 2. Materials and Methods

This study involves analysis of de-identified Electronic Health Record (EHR) data via Mayo Clinic Platform Discover. Data shown and reported in this manuscript have been extracted from the EHR using an established protocol for data extraction, aimed at preserving patient privacy. The data have been determined to be de-identified pursuant to an expert’s evaluation, in accordance with the HIPAA Privacy Rule. Any data beyond what are reported in the manuscript, including but not limited to the raw EHR data, cannot be shared or released due to the parameters of the expert determination to maintain the data de-identification. Contact corresponding authors for additional details regarding Mayo Clinic Platform Discover.

**Figure 1:**
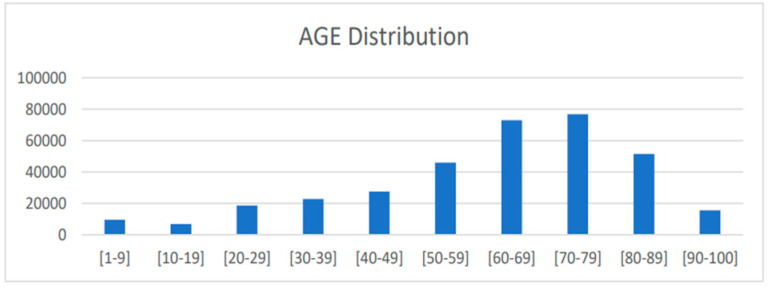
Age distribution in the cohort.

**Figure 2:**
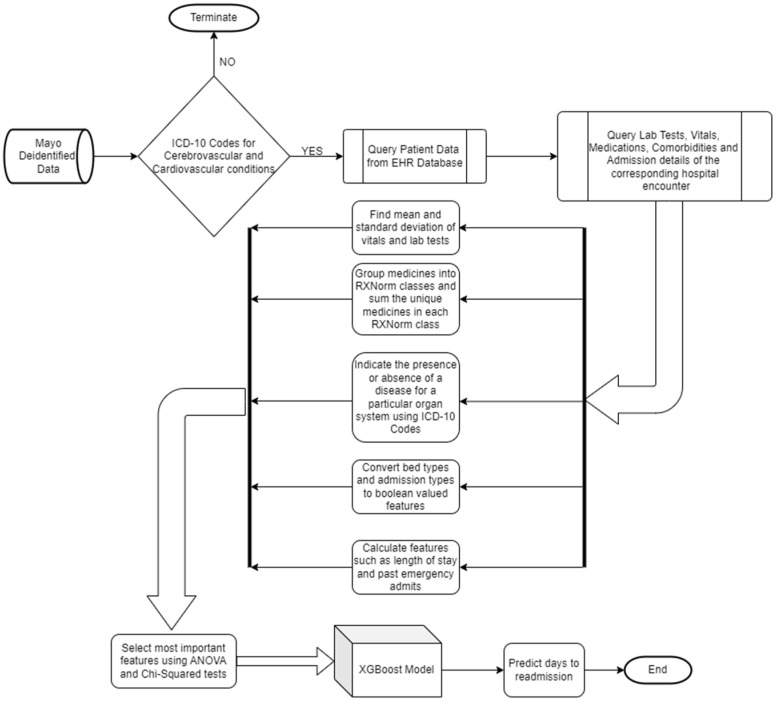
Methodology flowchart.

A sub-cohort was created using the above dataset with Epic systems as a data source, and hospital encounters with duration of stay greater than a day or an overnight stay with specific ICD-10-CM codes, encompassing conditions such as cerebrovascular disease, cerebral infarction, transient ischemic attack, heart and cardiac disease, carotid disease, migraine, and hypercoagulable states. Any encounter that was followed by a patient death within 7 days was dropped from the cohort. Approximately 310,000 encounters make up the cohort’s population dataset, which includes information on the patient’s age, gender, height, weight, smoking and alcohol status, admit–discharge settings, lab results, vital signs, medications, visit types, bed types, process types, and diagnosis. The cohort’s male population was roughly 53%, and the age range was highly divided, peaking between 60 and 80, as shown in Figure 1. The methodology flowchart, as depicted in Figure 2, shows details about how the data were collected, processed, and further used for training the model.

Vital signs and laboratory test results serve as critical indicators of a patient’s condition in a hospital setting, providing essential data to assess whether the patient is maintaining a stable state or experiencing health complications. This can be extended to understand readmission where a slight fluctuation in one of the lab results or vitals needs immediate attention. In this study, we averaged the lab test results and vitals and also calculated the standard deviation for the same. Laboratory test results such as serum sodium, serum potassium, hemoglobin, blood glucose, and blood urea nitrogen and vitals such as pulse rate and mean arterial pressure (MAP) were considered in this study.

ECG tests were also included in the study. The classification of the ECG results into various categories focused primarily on identifying “Normal” ECG readings and instances of “Atrial Fibrillation”. For additional ECG abnormalities, additional classifications were used. The ECG results were label-encoded based on the test results. Atrial fibrillation, in particular, is of significant clinical interest due to its documented direct correlation with cardiovascular disease risk, stroke, and all-cause mortality, as supported by this study’s [27] results.

To facilitate the analysis of our cohort and for training purposes, the medications administered during the patient’s hospitalization were systematically categorized into standardized RX Norm classes. The number of unique medicines administered in each RX Norm class was summed, and these classes were used as features for training the model.

Primary diagnosis codes and diagnosis codes present upon admission were used in the study. The diagnosis codes were transformed into feature vectors, each of which represented the specific organ system or physiological area associated with a distinct disease code. For instance, a code like I25.2, indicating a previous myocardial infarction, was interpreted as indicative of a circulatory system-related issue. Furthermore, the study took into account comorbidities that contribute to the calculation of the Charlson Comorbidity Index [9] as separate features.

Other features used in this study encompassed a wide array of variables that encapsulated the complexities of patient admissions and discharges. These features comprised admission types, bed types, patient process types, admit source, length of stay, the number of emergency visits in the previous six months, and discharge disposition. Each of these variables played a crucial role in characterizing the patient’s journey through the healthcare system. The data were transformed in order to prepare them for analysis and model training. The data were specifically transformed into Boolean-valued features. Each feature in this transformation had a binary value that was set to “True” if the related service or condition had been utilized or met. For instance, if an ICU bed was used while the patient was admitted, the corresponding feature for ICU admittance was set to “True”. The service or condition was given a “False” value, on the other hand, if it did not apply.

In order to identify and retain the most significant features for our analysis, we employed statistical techniques, specifically Analysis of Variance (ANOVA) [28] and Chi-Squared tests [29]. These tests were conducted to evaluate the relationship between the features and the target variable, such as readmission risk. Features that exhibited a *p*-value exceeding 0.05, signifying a weaker statistical relationship with the target variable, were judiciously eliminated from consideration. The resulting feature selection process led to a refined set of 150 features that were deemed highly relevant and statistically significant for our analysis.

A crucial step in assuring the accuracy and dependability of data for readmission [25] analysis is to handle missing values. In our study, the cohort presented various features with missing values, necessitating a systematic approach to address this issue. To streamline the cohort, all features with missing values that accounted for less than 1% of the cohort were selectively removed. Subsequently, a pooled regression method was applied to impute missing values for laboratory test results and vital signs, following the removal of any outliers in the dataset.

The dataset was divided into seven separate groups, each of which corresponded to a different period of time after the index admission for readmission [26]. The following temporal categories were chosen: “class 0” for readmissions occurring within 7 days or less; “class 1” for readmissions occurring between 7 and 30 days; “class 2” for readmissions occurring between 30 and 60 days; “class 3” for readmissions occurring between 60 and 120 days; “class 4” for readmissions occurring within 120 to 180 days; “class 5” for readmissions occurring within 180 to 365 days; and “class 6” for those exceeding 365 days.

**Figure 3:**
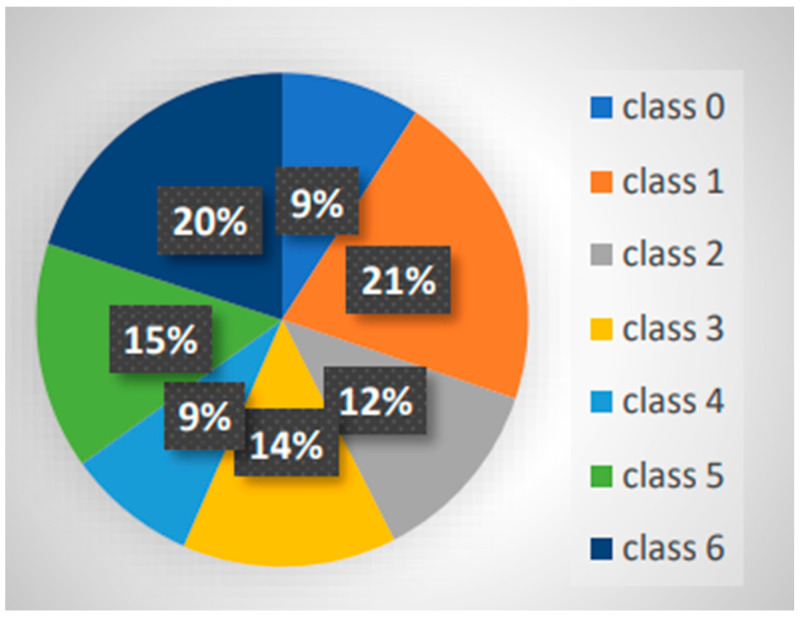
Distribution of data in each readmission class.

The machine learning model was trained using 80% of the data, with the remaining 20% being set aside for assessing its performance, according to the conventional 80–20 train–test data split. It is important to note that the test data were randomly chosen from the cohort in order to preserve the inherent data distribution within the cohort in the test subset. Furthermore, the dataset showed an intrinsic class imbalance as shown in Figure 3. Random under-sampling technique was used for the majority classes to balance out the representation of various readmission times in order to reduce this imbalance. This preprocessing step sought to reduce bias towards the majority classes while improving the model’s capacity to learn from the data.

Following the class balance adjustment, the data underwent a standardization process using a standard scaler. This scaling approach was crucial for ensuring that all dataset features were scaled uniformly, enabling a more reliable and precise machine learning model training process.

**Figure 4:**
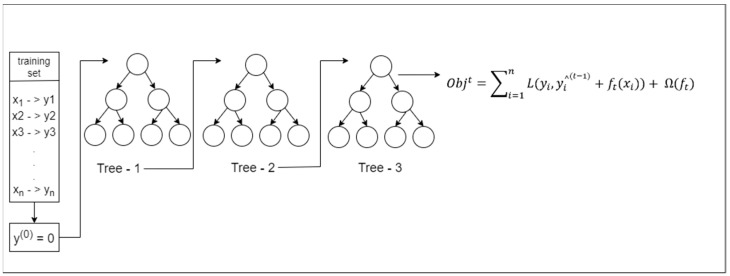
XGBoost architecture.

The model was trained using XGBoost algorithm. The model architecture is depicted in Figure 4. The main objective of the XGBoost algorithm is to minimize the loss $L^{\left(t\right)}$ as represented in Equation (1),

(1) $$L^{\left(t\right)}=\sum_{i=1}^{n}l\left(y_{i},\;{\hat{y}}_{i}^{\left(t-1\right)}+f_{t}(x_{i})\right)+\Omega (f_{t})$$

(2) $$\Omega \left(f_{t}\right)=\gamma T+\frac{1}{2\lambda {\left|w\right|}^{2}}=\gamma T+1/2\lambda \sum_{j=1}^{T}w_{j}^{2}$$

where $f_{t}$ represents the set of base learners. Ω is the regularization function. $x_{i}$ is the *i*th input to the model among n training points. $y_{i\;}$ is actual label of the *i*th input, and ${\hat{y}}_{i}^{\left(t-1\right)}$ is the output predicted by the (*t* − 1) base learner.

Let

(3) $$g_{i}=\frac{\partial L(y_{i},\;{\hat{y}}_{i}^{\left(t-1\right)})}{\partial {\hat{y}}_{i}^{\left(t-1\right)}}$$

(4) $$h_{i}=\frac{\partial^{2}L(y_{i},\;{\hat{y}}_{i}^{\left(t-1\right)})}{\partial {{\hat{y}}_{i}^{\left(t-1\right)}}^{2}}$$

Now, by using Taylor series expansion and expanding up to 3 terms, Equation (1) can be re-written with the help of (2), (3), and (4) as (5).

(5) $$L^{\left(t\right)}=\sum_{j=1}^{T}\left[\left(\sum g_{i}\right)w_{j\;}+\;1/2(\sum h_{i}+\lambda )\sum_{j=1}^{T}w_{j}^{2}\right]+\gamma T$$

Let $G_{j}$ = $\sum g_{i}$ and $H_{j}$ = $\sum h_{i}$

Equation (5) changes to

(6) $$L^{\left(t\right)}=\sum_{j=1}^{T}\left[G_{j}\;w_{j}+\frac{1}{2}\;(H_{j}+\lambda )w_{j}^{2}\right]+rT$$

Solve Equation (6) and find the best $w_{j}^{*}$ that minimizes the loss $L^{\left(t\right)}$. The next results correspond to the following:

(7) $$w_{j}^{*}=-\frac{G_{j}}{H_{j}+\lambda \;}$$

(8) $$L=-\frac{1}{2}\;\sum_{j=1}^{T}\frac{G_{j}}{H_{j}+\lambda }+rT$$

Here, Equation (7) represents the best $w_{j}^{*}$ and Equation (8) provides the total loss computed while training the XGBoost model.

Hyperparameter tuning was used to optimize the model’s performance and make it more accurate. The selected hyperparameters included the maximum depth of the decision tree (max_depth), subsample ratio (subsample), regularization lambda (reg_lambda), regularization alpha (reg_alpha), minimum child weight (min_child_weight), gamma, column subsampling at the tree level (colsample_by_tree), column subsampling at the level (colsample_by_level), and the maximum step size for updates (max_delta_step). The hyperparameters were selected using Grid Search and Bayesian Optimization. The parameters were selected based on their performance on the validation data.

## 3. Results

The model was trained in a distributed environment of 40 CPUs with 256 GB RAM. The XGBoost library implementation in Python was used for training the model. The objective function of the model was set to “multi:softmax”. 

**Figure 5:**
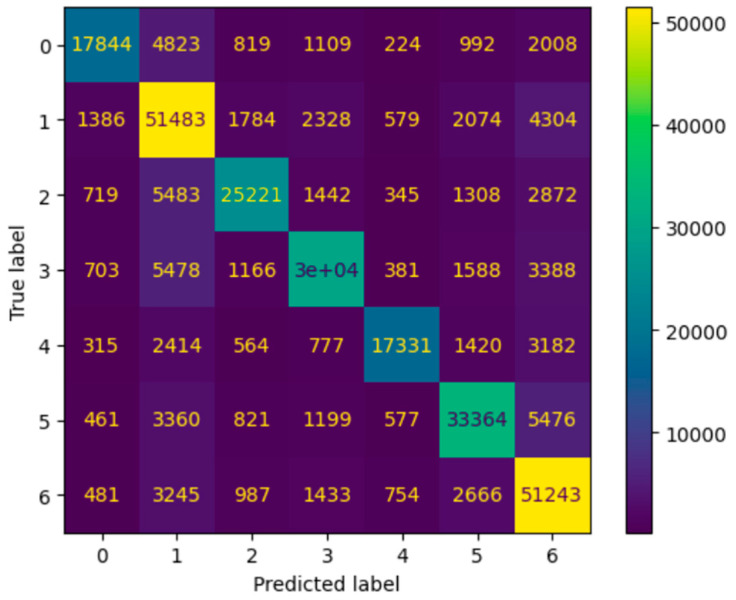
Confusion matrix of the proposed readmission risk model.

The main metric used to evaluate the model was accuracy, although precision and recall were also used to evaluate its robustness. The model was able to attain a precision score of 0.74 (±0.03) and recall score of 0.75 (±0.02). The model was close to 82 percent accurate (±5%). This was calculated by a weighted score in order to account for the imbalance of the data. Figure 5 shows the confusion matrix obtained by the model on the entire dataset. The model was able to identify 78.39 percent of the patients who were readmitted within 30 days, 72.20 percent of the patient readmissions within a 30–120-day timeframe, and 80.81 percent of the patient readmissions after 6 months.

## 4. Discussion

The model was able to predict more accurately than the LACE plus score. For the patients who were admitted within 30 days, only 47.70 percent of the patients had a LACE plus score of greater than or equal to 70. Our model was able to accurately identify 78.39 percent of such patients. For those patients with greater than six months readmission, only 10.09 percent of such patients had a LACE plus score of less than or equal to 30. Our model was able to identify 80.81 percent of such patients accurately.

**Figure 6:**
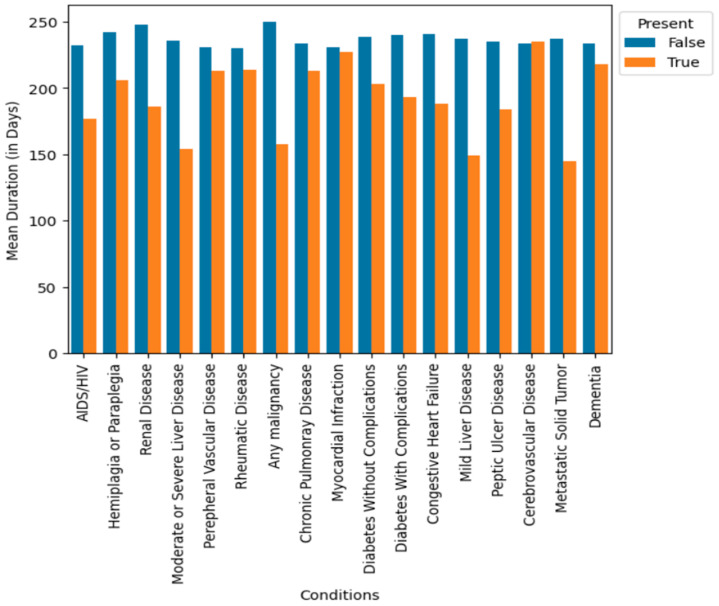
Mean duration between readmission when certain comorbidities were present. Here, ‘True’ represents the mean of patients with the particular comorbidity, while ‘False’ represents the absence of that comorbidity.

Our research yielded several significant insights when examining the risk of readmission. Those patients with a higher burden of comorbidities were more likely to experience readmissions, particularly when they had conditions such as renal disease, liver disease, or tumors, which were associated with the highest risk of readmission, as indicated in Figure 6. This observation highlighted a strong positive correlation between the Charlson Comorbidity Index (CCI) and the likelihood of readmission. Other diseases such as rheumatic disease, chronic pulmonary disease, myocardial infarction, and dementia do not significantly impact the number of days to readmission. Those patients with an absence of comorbidities, as reported in Figure 6, have decreased lengths of stay in the hospital when compared to the presence of a comorbidity. Specifically, those patients affected by hemiplegia or paraplegia tend to have longer stays in the hospital, with a mean of 24 days, while those patients with other comorbidities have a mean length of stay between 6 and 12 days. The patients diagnosed with neoplasms and hematologic diseases tend to be readmitted approximately 40% sooner compared to those without such conditions.

**Figure 7:**
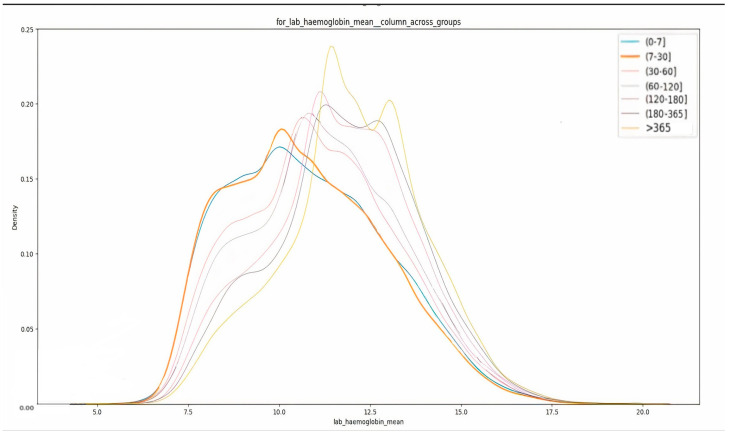
Distribution of hemoglobin values across different readmission risk classes.

The cohort showed that the patients with hemoglobin levels below the normal range exhibited a higher rate of readmission, as shown in Figure 7. This finding underscores the significance of monitoring and addressing low hemoglobin levels as a potential risk factor for readmission. The observation that the patients who were prescribed a higher number of medications during their hospital stay experienced shorter times to readmission underscores a crucial relationship between medication management and readmission risk. This finding aligns with and reinforces the results obtained in [8]. The patients admitted to the overflow bed type had a mean readmission duration of approximately 120 days, whereas those who were not admitted to the overflow beds had a mean readmission duration of about 235 days.

## 5. Conclusions

In conclusion, a robust readmission risk assessment model was developed using the Mayo Clinic dataset. The model proved to be highly effective in predicting the risk of readmission for individual patients at the time of their discharge. The unique classification model introduced in this study stands out for its distinctive approach. Instead of predicting whether a patient will be readmitted within a specific timeframe, this model is designed to predict the actual timeframe of the readmission. This innovative feature broadens its applicability by encompassing the readmission risk for multiple conditions rather than focusing solely on specific disease-to-disease scenarios. This study could be extended to cohorts with a broader range of diseases beyond cardiovascular and cerebrovascular conditions. Incorporating disease progression as a feature would improve the accuracy of readmission predictions. Additionally, including specific medications and their dosages, rather than general RXNorm classes, would enhance the model’s predictive capability.

The practical implications of this model are significant. It enables healthcare providers to make more informed clinical decisions by providing a precise estimate of the expected time to readmission for each discharged patient. This level of granularity empowers healthcare professionals to tailor care plans to individual patient needs as the model indirectly indicates the risk profile of the discharged patient based on the predicted number of days to readmission. Consequently, differential care can be administered to optimize the patient outcomes and healthcare resource allocation. This model represents a valuable addition to the healthcare landscape, enhancing the ability to deliver personalized patient care and improve the overall quality of healthcare services.

## Data Availability

This study involves analysis of de-identified Electronic Health Record (EHR) data via Mayo Clinic Platform Discover. Data shown and reported in this manuscript were extracted from the EHR using an established protocol for data extraction, aimed at preserving patient privacy. The data were determined to be de-identified pursuant to an expert’s evaluation, in accordance with the HIPAA Privacy Rule. Any data beyond what are reported in the manuscript, including but not limited to the raw EHR data, cannot be shared or released due to the parameters of the expert determination to maintain the data de-identification.
